# Improved meals service and reduced food waste and costs in medical institutions resulting from employment of a food service dietitian – a case study

**DOI:** 10.1186/s13584-020-0362-0

**Published:** 2020-02-03

**Authors:** Orit Yona, Rebecca Goldsmith, Ronit Endevelt

**Affiliations:** 1 Nutrition Division, Ministry of Health, Jerusalem, Israel; 2 School of Public Health, University of Haifa, Haifa, Israel

**Keywords:** Food service, Dietitian, Hospitals, Food costs, Food waste

## Abstract

**Background:**

A recurring problem in medical institutions is patients not always receiving food meeting their nutritional and medical needs. A proposed contributing factor is non- inclusion of dietitians in food service staff. Recently, positions for food service dietitians in hospitals were created. For the newly defined role of “Food Service Dietitian”, comprehensive training courses were developed (70 dietitians participated).

**Objective:**

To examine the impact of the addition of the role of a “Food Service Dietitian” in medical institutions on suitability of foods served, food costs and food waste.

**Methods:**

A three years (2014–2017) national case study to examine the new role’s impact was carried out, in 18 hospitals, nine of which employ a food service dietitian (intervention), and 9 without (control). The number of nutritional analyses of menus was checked, as was the extent of kitchen staff training, and how often night meals were served for all patients. Data were gathered regarding food costs and waste with respect to food distributed to staff and patients. Food costs savings and waste reduction were calculated, based on reduction in provision of unnecessary meals, at a cost of 18 NIS per day per meal.

**Results:**

Kitchen staff training was carried out in all intervention institutions, and not in the controls. In most controls, nutritional analyses were not performed, whereas in the intervention hospitals, full analyses were performed and tailoring of menus to specific department requirements improved significantly. In most intervention hospitals, late night snacks were provided, this not being so in the controls. Total food cost savings of $229,569 per annum was seen in the six intervention hospitals, attributable to 4 factors:

1. Meals not delivered to fasting patients, or those receiving parenteral/enteral nutrition- cost savings of 328,500 NIS ($93,857)
2. Better tailoring and monitoring of food delivered to the wards and staff (bread, cheese, milk etc)- annual cost savings of 235,000 NIS ($67,142) in the hospitals with a food service dietitian.
3. Checking expiry dates of medical foods, and improved communication between the wards, the kitchen and the food distribution centers, has lessened food waste with savings of 5% from the medical food budget per annum of 40,000 NIS ($11,428).
4. As a result of dietitian-performed nutritional analyses, tailoring of food provided according to the patient’s medical and nutrition needs was improved. In one hospital, after re-evaluation of serve sizes in high protein diets, sizes were reduced while retaining adequacy, with immediate cost savings of 200,000 NIS ($57,142) per annum.

**Conclusions:**

Implementation of the new role of Food Service Dietitian led to cost savings and significant improvements in adherence to the nutritional care plan.

## Introduction

Among hospitalized patients, there are many complex and varied illnesses, which, according to the medical condition and nutritional status of the patients, require tailored treatments concerning types of food, their composition, preparation, texture, and the manner of serving [1]. Serving food appropriate to the patients’ medical needs is a critical component of medical and nutrition care, as this promotes and improves health, treats illness, and leads to faster recovery of hospitalized patients.

Data collection and findings of audits and supervisory visits to medical institutions and processing by the Nutrition Division at the Ministry of Health in Israel show that the continuity of care between the hospital departments and the hospital food service to be reflected in serving food appropriate to the patients’ medical needs is incomplete or even nonexistent in some hospital departments.

Implementation of the nutrition program by the food service has encountered many difficulties, due to the inability of the kitchen and the entities that link the food service to the departments to implement the program. Thus, often the patients do not receive the optimal food for them according to the medical and nutrition guidelines issued in the department even if they have the right nutritional instructions.

Several years ago, in order to define the problems and to propose solutions, the Nutrition Division in the Israel Ministry of Health initiated extensive discussions. One of the research-based solutions was based on the experience in the United States of integrating dietitians in the position of Senior Coordinator Nutrition and Food Control [2]. This position was created in order to improve the situation and ensure the provision of nutritious food to the patient [3], throughout the food chain: from supervision and quality control of raw materials prior to purchase, storage, production of dishes, conservation processes, transport and delivery to patients according to their medical condition and to reduce food waste.

It was hypothesized that hospitals that implemented the change i.e. employed a Food Service Dietitian, will have better continuity of nutritional treatment between foodservices and medical wards and patients will receive food according to their medical condition and nutritional treatment plan as compared with the control hospitals.

The Ministry of Health’s policy is “patient-centered” and this is expressed by use of a multi-disciplinary approach, ensuring personalization of treatment, patient safety, and accessibility. Part of this approach involves nutritional support and diet therapy and in particular, personalization of the menu offered, in accordance with medical indications and personal preferences. One outcome of the routine Ministry inspections was that it was observed that there was often a lack of correlation between the medically defined needs on the one hand, and the food offered and served on the other hand. As a result, the following course of action was implemented 1. Together with the Civil Service Commission, clarification of the role of the food service dietitian and tasks involved 2. Assessment of the function of a food service dietitian by carrying out a case study comparing various outcomes in hospitals with a food service dietitian as compared to those without 3. Implementation of a training course for the role| 4. Updating of the directives regarding hospital food in general and the role of the food service dietitian in particular.

## Methods

The Nutrition Division at the Israeli Ministry of Health defined and developed, together with the Ministry’s Human Resources Department, a new occupation role, which was recently approved by the Civil Service Commission of Israel. The role was defined as “Food Service Dietitian”. The necessity for formal definition of this new role arose following revision of the Ministry of Health directives for feeding of patients in hospitals, when it became clear that there was an imperative need for dietitians to be far more actively involved in the tailoring of menus and delivery process of food in all of its aspects.

A three years (2014–2017) national case study to examine the new role’s impact was carried out, in 18 hospitals, 9 of which employ a food service dietitian (intervention), and 9 without (control).

In the years 2014–2017, 18 general hospitals were inspected and audited by the Ministry of Health, and for the purposes of the case study, 9 with a food service dietitian, and 9 without were selected.

1. A comprehensive course was developed to train dietitians for this role with all its related tasks. This training is an essential prerequisite for working in this role. The training included issues of: sanitation, food safety, planning and writing institutional menus, including according to medical diagnoses, healthy cooking, defining food preparation processes, methods of control in the kitchen and in medical department and wards. The dietitians working in the intervention hospitals had all undergone the training course.
2. Some hospitals employed dietitians who had completed the training course in the new role.
3. Costs of meals, and potential costs savings as a result of employment of food service dietitians, were calculated. This took into account the costs of employment of the food service dietitians.
4. The Ministry of Health performs a periodic audit of all general hospitals. Licensing, including renewals, is dependent on the audit outcomes. The purpose of the audit is to improve the quality of care.
5. As part of these audits, a three-years (2014–2017) national case study to examine the new role’s impact was carried out, in 18 hospitals, 9 of which employ a food service dietitian (intervention), and 9 without (control). The impact of the food service dietitian was assessed according to the following parameters:
  - Training of food service staff in current nutrition guidelines for hospitalized persons. These are uniform for all general hospitals, are specified in detail in Ministry of Health Directives, and take into account patient mix.
  - Nutritional analysis of menus performed
  - Night meals served to the patients, as is required according the Ministry of Health Medical Administration Directive
  - Food costs savings and waste reduction were calculated

## Results

### Training course and subsequent employment of dietitians in the new role

Seventy dietitians completed a special training course, organized by the Nutrition Division, Ministry of Health, for this role. The training course focused on the supervision of food services in medical institutions.

The dietitians who fulfill this role all completed the training course. This specially designed course is necessary, as it teaches and focuses on topics not taught in the undergraduate and practical nutrition training programs. The training for this position focuses on the following topics: food safety, work interfaces on the one hand with food system managers and cooks, and on the other hand, multi-professional teams in the inpatient departments. In addition, the course teaches how to perform a nutritional assessment of the inpatient wards for each ward according to its diagnoses and medical conditions in order to build and implement an appropriate menu for the ward in general and for each patient in particular according to medical and dietary needs.

The cost of training for the course is about NIS 2500 per participant.

1. Conducting on-going training of kitchen staff: In the control hospitals, except for one where there is some partial training, there is no training of kitchen staff. In all of the hospitals with a food service dietitian, training of kitchen staff is carried out. Shown in Fig.1.

**Fig. 1:**
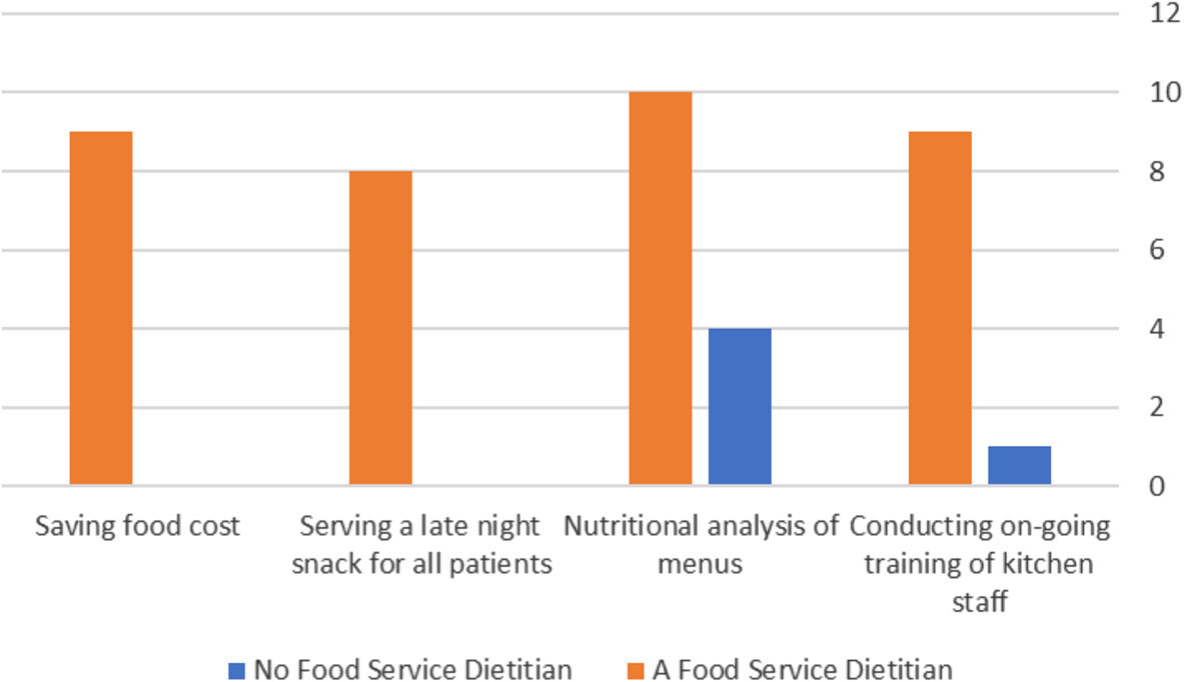
Hospitals achieving the implementation of the following parameters
2. Nutritional analysis of menus: In all of the hospitals with a food service dietitian, full nutritional analyses of the menus are carried out. In 55.5% (5) of the control hospitals, there are no nutrition analyses of the menus, with partial analysis carried out in 3 (33.3%) of the control hospitals and only in one (11.1%) are full analyses carried out. (Shown in Fig 1).
3. Serving a late-night snack for all patients: In the 8 (88.8%) hospitals with a food service dietitian, late night snacks are always served as required. In only one (11.1%) of the control hospitals, late night snacks were served.
4. Food cost savings – shown in Table 1 below
5. Special meals (such as low sugar, low fiber, low lactose, low potassium) are provided, as is documented in the Ministry of Health inspections, but data were not collected as to changes or improvements in provision of special meals.

**Table 1:** Financial savings in six hospitals following a computerized application for notification of meals needed between the inpatient departments, and the food service

Hospital	Number of EN,PN feedings daily “SAVED”	Annual savings in NIS (Shekels)	Annual savings (in Dollars) * exchange rate 3.5NIS = $1)
Hillel Yaffe Medical Center	50	328,500	93,857
Assaf Harofeh Medical Center	50	328,500	93,857
Poriya Medical Center	35	229,950	65,700
Wolfson Medical Center	50	328,500	93,857
Sheba Medical Center	60	394,200	112,628
Tel Aviv Medical Center	60	394,200	112,628
Total	305	2,003,850	$572,527

Six out of nine hospitals included in the intervention implemented the computerized application. (*EN* Enteral Nutrition, *PN* Parenteral nutrition)

### Economic impact of the process

There were significant cost savings, resulting from reduced food waste, as a result of better suitability and control of food served according to the medical and nutrition needs of the patient.

### Subjective observations

As a result of the food service dietitians’ employment, the dietitians in this new role reported the following achievements:

- Implementation of basic principles of healthy nutrition, to ensure that food provided in medical institutions will meet the nutrition needs of the hospitalized patients.
- Improvement in the taste and variety of food served.
- Careful attention to food safety along the food chain- from before purchase of raw ingredients, storage conditions, meal production, standardized recipes, and ending with manner of serving of food, and consumption.
- Working according to standard recipes to enable standardization and maintenance of consistent nutrient content of foods.
- Training of food service staff in the following relevant aspects of food service management: nutrition principles, storage conditions, production methods, food preservation, distribution by accurate size portions, quality control and aspects of transport of food to the patient.
  - **Cost savings were assessed according to the following parameters:** In six of the medical centers where the intervention took place, a computerized application for communication between the inpatient departments and the food service was developed, which allows for computerized continuity and documentation of treatment. This meant that in cases where a patient receives either Parenteral Nutrition (PN) or Enteral Nutrition (EN), a normal diet would not be ordered. Prior to the computerized communication, in many cases regular meals were ordered for these patients but not consumed, as the patients’ condition necessitated enteral or parenteral nutrition. This improvement results in a savings of 328,500 NIS ($93,857) per annum, as patients with enteral or parenteral nutrition did not receive meals they could not consume.
    Method of “savings” calculation: The average daily cost per patient of a normal menu, consisting of three meals a day and a late evening snack is 18 NIS ($5.14), based on figures obtained from general hospitals. Since the costs of kitchen staff and overheads are fixed, the savings are only related to the food costs. Thus, based on an average of 50 enteral and parenteral nutrition feedings daily, the savings is 900 NIS/day and 328,500 NIS per annum. This is shown in Table 1, according on the number of enteral and parenteral nutrition feedings daily in each of the six hospitals where the computerized system was in place and operational**.**
  - The supervision by means of observations and random checks of amounts distributed to the various inpatient wards and staff, of foods such as milk, cheese, dairy products, sugar, artificial sweeteners, salt and bread, has resulted in a decrease in the amounts distributed, leading to an immediate annual savings of 235,000 NIS ($67,142) in the hospitals with a food service dietitian. One example is checking of the method in which bread is distributed and a request to finish one loaf before opening another.
  - As a result of dietitian-performed nutritional analyses, tailoring of food provided according to the patient’s medical and nutrition needs was improved. In a 1500 bed hospital (Sheba) after re-evaluation of serve sizes in high protein diets, sizes were reduced while retaining adequacy, with immediate cost savings of 200,000 NIS ($57,142) per annum.
  - Checking the expiry dates of medical foods, and improved communication between the wards, the kitchen and the food distribution centers, has lessened food waste and has resulted in a savings of 5% from the budget for medical food per annum, equivalent to 40,000 NIS ($11,428).
  - In one of the biggest hospitals, switching from centralized serving at the ward level to each patient receiving a personalized tray prepared by the kitchen, resulted in an overall 25% reduction in the number of portions, resulting in an immediate savings of 104,000 NIS ($29,714) per annum.

In total, the cost savings of these 4 steps (b to e) in hospitals where there are food service dietitians have been about 579,000 NIS ($165,428) per annum in the seven hospitals surveyed. All hospitals carried out steps b to e, but only 7 of the 9 fully documented the cost savings (Table 2).

**Table 2:** The cost savings of these 4 steps (b to e) in 7 of the hospitals surveyed, where there were improvements in processes as a result of the work of a food service dietitian

The process	Annual savings in NIS (Shekels)
Adjustment of orders of staples such as: bread, milk etc	235,000
Adjustment of protein portion sizes	200,000
Checking the expiry dates of medical foods, and improved communication between the wards, the kitchen and the food distribution centers	40,000
Switching from centralized serving to offering of personalized trays	104,000
Total	579,000 ($165,428)

### Calculating the cost-effectiveness of the role of food service dietitian

1. Total financial savings in six hospitals following introduction of a computerized application (Table 1**)** for notification of meals needed between the inpatient departments, and the food service is 2,003,850 NIS ($572,527).
2. The total cost savings of the 4 steps (b to e), in hospitals where there are food service dietitians, have been about 579,000 NIS ($165,428) per annum in the 7 hospitals surveyed.
3. The cost of a dietitian for a year is 150,000 NIS ($42,857). This includes social overheads such as national insurance, pension contribution and other costs. The sum of the financial savings is: 1,532,850 NIS.
4. According to the following calculation: (2,003,850 + 579,000)- (150,000*7) = 1,532,850 NIS ($437,957)

The total estimated cost savings for the 7 hospitals surveyed, as a result of filling this position, is 1,532,850 NIS ($437,957), generated by improved processes, as shown above.

## Discussion

Most inpatients depend on the hospital menu to meet their nutritional needs and adequate nutrition is especially important in the acute care hospital setting where 20–50% of patients are malnourished [4]. Advances in technology enable patients to order their meals using a bedside computerized ordering system, instead of having the order hand written [5, 6]. In some of the medical centers included in our study, a computerized application for communication between the inpatient departments, and the food service was developed, which allows for computerized continuity of treatment. This meant that in every case where a patient receives either Parenteral Nutrition (PN) or Enteral Nutrition (EN), a normal diet would not be ordered. Prior to the computerized communication, in many cases regular meals were ordered for these patients. This improvement, even in the first stage, results in a savings of 328,500 NIS ($93,857) as patients with enteral or parenteral nutrition ceased receiving meals they could not consume.

In this study, there is an underestimation of the financial savings resulting from implementation of the computerized communication between inpatient departments and the food service (kitchen). This is as a result of patients not consuming meals as they are fasting, not wishing to eat (nausea/pain), post-operative, or undergoing tests. The calculations presented in this study refer only to the savings resulting from meals not provided because of EN and PN feedings. If the costs of meals ordered and sent, but not consumed, for reasons mentioned were factored in, then the savings would be even greater.

Another important issue requiring attention is that of plate waste, defined in the hospital setting as “served food that remains uneaten by patients” [7] and it has been reported to be up to 67% in hospitals [8]. Significant plate waste reduces the likelihood of patients meeting their nutritional requirements [9], leading to poorer clinical outcomes and increased hospital costs [10].In our study as a result of the work of the food service dietitians there was reduction of plate waste, resulting from better tailoring of portion sizes to patient needs and ages (medical diagnoses, children, infants, adult etc.). At the Sheba Medical Center (1500 bed hospital) an adjustment by a food service dietitian of the protein portion sizes to the nutritional needs of the patients was done, resulting in an immediate savings of about 200,000 NIS ($57,142) per annum.

The British Dietetic Association published the results of an audit which demonstrated that most patients are not meeting the nutrient standards recommended by the BDA Nutrition and Hydration Digest. Recommendations include the provision of energy/protein- dense snacks and menus, and offering Oral Nutrition Supplements (ONS) where clinically indicated, in addition to training of staff. A food services dietitian is ideally placed to lead this, forming a vital link between patients, caterers and clinical teams [11].

In our periodic audits of all general hospitals, carried out by Ministry of Health staff, we found that in hospitals where there is a food service dietitian, all the following was done: monitoring and checking of all menus including: ingredients, serve sizes, nutritional analyses of menus,method of serving of food, all in accordance with the Ministry of Health Medical Administration Directive. In addition, the audits showed that monitoring of all food preparation processes, from checking raw products, calculating nutritional values of foods prepared, served, and full documentation of all steps was carried out, as was serving a night meal for all patients.

Another study [12] investigated if a protein-enriched menu in conjunction with individualized dietary counseling by a clinical dietitian would increase energy and protein intake in hospitalized patients at nutrition risk as compared with providing the protein-enriched menu as a stand-alone intervention. This study concluded that providing a protein-enriched menu in conjunction with individualized dietary counseling by a dietitian significantly increased protein and energy intake in those hospitalized patients at nutrition risk. It showed that 90% of the included patients reached at least 75% of their energy and protein intake. This can be an effective strategy to combat under-nutrition in hospitals, including both a protein-enriched hospital menu and dietary counseling by a clinical dietitian. In our observations during audits we found that in hospitals where there is a food service dietitian, the patients receive the foods they need in accordance with their medical and nutrition status, and this is essential as part of the therapy and recovery of the patient.

We consider that in order to create a proper sequence of treatment, the role of a Food Services Dietitian is essential, to ensure that there is an accurate translation of the dietitian’s inpatient nutritional treatment plan given in the wards to the food produced and served by the hospital’s food service to each patient.

The importance of this role “Specialist Dietitian in Food and Beverage Management Services” and its contribution was also reported by the BDA (British Dietetic Association), as Food Service Dietitians belong to a BDA Specialist Group, the aims of which are to raise inter-agency awareness of the vital, comforting and enjoyable role played by patient food and beverage services in all public sectors, particularly in health and social care settings. The group works across professional boundaries to highlight the importance of services that are designed, operated and focused on meeting the nutrition and hydration needs of the clients served [13].

Our findings reinforce these findings and show that there should be a specialized role for a dietitian to liaise between the medical and nutrition instructions given in the wards and the food served by the food service.

Hospital foodservice is increasingly focused on not only improvement in levels of patient’s satisfaction and cost savings, but also on influencing clinical outcomes associated with nutritional intake.

A recent study, in 2018, published comprehensive measurements of outcomes reported for RS (Room Service) in a public hospital setting, where RS allows patients to order meals ‘for a time that suits them’ which meet their nutritional requirements. Meals are prepared and delivered within 45 min of an order being placed [14]. This study found statistically significant increases in both total energy and protein intakes, with the implementation of RS compared to TM (traditional foodservice model). These positive outcomes including reported cost benefits are important to hospitals that continue to investigate strategies to assist patients to increase their nutritional intake, as poor food intake has been recognized as a risk factor for negative and costly clinical outcomes and an increase in nutritional risk [14]. The involvement of a Food Service dietitian in this study was significant and important to the operation and success of this study, further emphasizing the need for this role.

In hospitals in the United States, this role has been known for many years. The position paper of the Academy of Nutrition and Dietetics details the recognition by food services of the vital importance of dietitians working jointly with catering and nursing colleagues, understanding that they are more effective together. According to the Academy, in foodservice systems, dietitians manage and direct foodservice operations in health care and other institutions and commercial settings or are employed in these capacities as employees of contract foodservice management companies (e.g., hospitals, schools, colleges and universities, continuing care communities rehabilitation centers, extended care settings, government facilities and correctional facilities), and commercial settings (restaurants, food vending and distribution, catering). Responsibilities include participating in managing, or directing any or all of the following: menu and recipe management food, supplies, and equipment purchasing; food receiving, storage reparation, and service; financial management; human resource management, food safety and sanitation programs; waste management, water conservation and composting programs, vending services and catering for special events; foodservice in emergency situations, and kitchen design. Registered dietitians use a wide variety of electronic tools to manage data and may specialize in the development and management of specific technological applications related to foodservice operations [15].

Our findings support the position paper of the Academy of Nutrition and Dietetics and emphasize the need for the role of a Food Services Dietitian.

Following completion of the study in 2017, its results were presented to management of general hospitals. Concurrently, an updated version of the Ministry of Health Medical Administration Directive [16] regarding food services was distributed. It listed the activities and responsibilities associated with this role, namely a Food Service Dietitian. In addition, during routine audits and inspections in institutions without a Food Service Dietitian, the therapeutic and economic advantages of employment of a Food Service Dietitian were presented. As a result, there is a growing number of institutions employing Food Service Dietitians. The intention is that in each medical institution, in accordance with size and number of patient/residents, a Food Service Dietitian will be employed.

Until now, 25 government and private medical institutions have filled this position. Most of the management of the general hospitals understood and recognized the importance of the role of the Food Service Dietitian, and allocated a position for this, from among the positions available.

The Food Service Dietitian also has various other tasks which include: supervision of nutritional quality of raw materials (foods) ordered; menu development, tailored to differing needs of inpatient departments (surgery, pediatrics, geriatrics, dialysis etc); supervision of food preparation, menu adaptation for individual patients where required: kitchen staff supervision, and more.

Food Service Dietitians will perform mapping and nutritional characterization of patients’ needs that includes at least consideration of the following aspects:

- Adjusting menus to the patient’s medical and nutritional status
- Serving a variety of fresh and cooked foods with high nutritional value with restriction of serving processed and ultra-processed foods.
- Ensuring that menus for patients with allergies and food sensitivities, such as Celiac disease, will be approved by the Food Service Dietitian.

The dietitian hired for this role must participate in the special training course for Food Service Deititian in order to fulfill this role.

### Innovation

The Food Service Dietitian is a new position in medical institutions in Israel. The impact of the innovation is in the following processes and areas:

- Cooperation and mutual learning, country wide
- Joining of forces of the Ministry of Health and general hospitals
- More efficient use of money
- Significant potential cost- savings
- Improvement of patient experience

A model, which can be replicated and integrated countrywide in all medical institutions and within catering companies.

### Future challenges- incorporating the “patient centered approach”

1. Enabling the individualized serving of food suited to the patient, according to determination of his medical and nutrition status
2. Enabling patients to select their food and time of meals, within the defined medical and nutrition parameters (“A personalized tray”)
3. Checking the effect of timing of patients’ meals in accordance with appetite and ability to eat (Room Service) on food intake and on clinical and non-clinical outcomes.
4. To check how much food is actually consumed and how much is served but not consumed and therefore thrown out.. This includes beverages also.
5. Checking what are the logistic changes required in the Food Service to accommodate timing of patients’ meals in accordance with patients’ appetite and ability to eat (Room Service)
6. Development of a book of standardized recipes
7. Completion of computerization of communication between hospital wards and the kitchen, in terms of meals and doctor’s orders
8. Improvement in levels of patient and staff satisfaction in relation to food provision
9. Monitoring by the Ministry of Health to examine patients’ satisfaction with nutritional treatment, including as a function of the work of the Food Service Dietitian

### Limitations

Patients receiving foods they need in accordance with their medical and nutrition status was assessed in observations rather than in the form of a random controlled intervention study. We did not check if most patients are meeting the nutrient intake standards recommended by the Nutrition Division in the Ministry of Health in Israel.

## Conclusions

In conclusion, the Food Service Dietitian’s supervision of the nutrition care sequence and involvement in the food service chain links and ensures communication between the medical and nutrition needs of the hospitalized patient, and the food needed, and ensures the necessary continuity of care. This role enables translation of the medical and nutrition needs into food preparation in the kitchen and guarantees that the patient receives his food in optimal conditions, and that the food is suited to his medical and nutrition needs. The achievements include also cost savings and reduction in food waste. The Food Service Dietitian can act as an extra precautionary factor in terms of infection prevention and food safety.

Based on the study results, the Nutrition Division in the Ministry of Health requires that every hospital have a Food Service Dietitian, with the scope (weekly hours) in accordance with institution size, patient numbers and needs. The Ministry of Health directive also supports this and requires that all menu types, including for staff, will be designed and supervised by a Food Service Dietitian.

## Data Availability

The datasets generated during and/or analyzed during the current study are not publicly available [Ministry of Health data for hospital renewal license audits], but are available from the corresponding author on reasonable request.
